# Are there species smaller than 1 mm?

**DOI:** 10.1098/rspb.2013.1248

**Published:** 2013-09-22

**Authors:** Axel G. Rossberg, Tim Rogers, Alan J. McKane

**Affiliations:** 1 Centre for Environment, Fisheries and Aquaculture Science (CEFAS), Pakefield Road, Lowestoft NR33 0HT, UK; 2 School of Biological Sciences, Queen's University Belfast, 97 Lisburn Road, Belfast BT9 7BL, UK; 3 Centre for Networks and Collective Behaviour, Department of Mathematical Sciences, University of Bath, Claverton Down, Bath BA2 7AY, UK; 4 Theoretical Physics Division, School of Physics and Astronomy, University of Manchester, Manchester M13 9PL, UK

**Keywords:** ecological species concept, lineages-through-time plots, adaptive dynamics, individual-based models, competition

## Abstract

The rapid advance in genetic sequencing technologies has provided an unprecedented amount of data on the biodiversity of meiofauna. It was hoped that these data would allow the identification and counting of species, distinguished as tight clusters of similar genomes. Surprisingly, this appears not to be the case. Here, we begin a theoretical discussion of this phenomenon, drawing on an individual-based ecological model to inform our arguments. The determining factor in the emergence (or not) of distinguishable genetic clusters in the model is the product of population size with mutation rate—a measure of the adaptability of the population as a whole. This result suggests that indeed one should not expect to observe clearly distinguishable species groupings in data gathered from ultrasequencing of meiofauna.

## 1. Introduction

The nature of the process of species formation has been the subject of debate since before the time of Darwin. Allopatric speciation through geographical separation is by now reasonably well understood, but the prevalence and mechanics of sympatric speciation are still a matter of debate [1]. Underlying this dilemma is the perhaps even more fundamental question of precisely what constitutes a species. For prokaryotic organisms, it is accepted that complications such as horizontal gene transfer pose a significant challenge to the traditional understanding [2]. Even in metazoa, the picture is not completely clear: the extreme biodiversity of meiofaunal organisms makes it difficult to distinguish between inter- and intraspecific genetic differences. In this paper, we will examine the results of a stochastic individual-based evolutionary model, comparing both with previous theory and with experimental data, building up an argument that the answer to the question posed in the title of this paper may be negative.

The species concepts we use here are the ‘genotypic clustering species concept’ [3, p. 296], below *genetic species* for short, where species are identified as distinct genotypic clusters, and van Valen's ‘ecological species concept’ [4, p. 233]. An ecological species, below *ecospecies*, ‘is a lineage (or closely related set of lineages) which occupies an adaptive zone minimally different from that of any other lineage in its range’ [4, p. 233]. Van Valen's ‘adaptive zone’ is essentially what is today called a niche, and it is understood that the boundaries of the niche may be defined either by the environment or be ‘imposed on it by the nature of the particular species that happen to be present together’ [4, p. 234].

In later years, the theory of adaptive dynamics has unveiled robust mechanisms by which evolving populations of sexually or asexually reproducing organisms can self-organize to form well-defined ecospecies [5,6]. These ecospecies have distinct phenotypes and are thought to be reproductively isolated, and so should be recognizable as genetic species in the sequence data. Mathematical models now have an important role to play in validating theories of speciation, with a large amount of data becoming available to test the diverse predictions of the various mechanisms of species formation.

In this paper, we will examine one such prediction made by a model consisting of a large number of organisms which undergo reproduction with mutation, and death through competitive interaction. The basic model concept goes back to MacArthur & Levins [7], but we have recently shown that the consideration of demographic noise present in an individual-based version of this model overturns some of the main theoretical findings [8,9]. The predictions of our analysis will be examined against observations from the emerging field of environmental genomics, where high-throughput sequencing technology is applied to analyse samples covering entire ecological communities. In several recent studies [10], these techniques were applied to study the biodiversity of meiofauna (or mesofauna), that is, of animals in the approximate size range of 0.05–1 mm.

**Figure 1.**
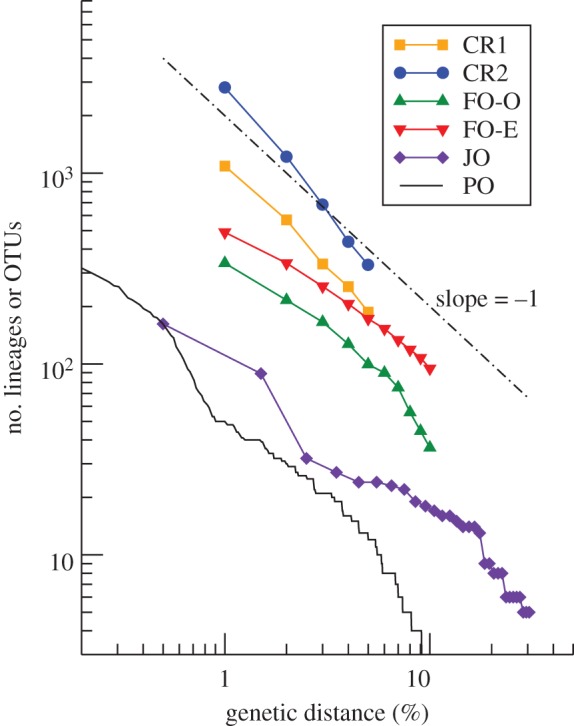
Empirical relationships between genetic distance and observed numbers of lineages or OTUs in environmental samples of meiofauna (upper four lines) and larger insects (lower two lines). Data as reported in the literature, see table 1. The dashed-dotted line represents the scaling relation (lineages)∼1/(distance) for comparison. (Online version in colour.)

According to a typical experimental protocol, organisms are extracted from fixated environmental samples, for example, by sieving, and then PCR-ready DNA samples extracted using standard laboratory techniques [10]. A selected region of the genome comprising a few hundred base pairs is then amplified by PCR with universal primers, and the amplicons sequenced using high-throughput technology. The output is a database of thousands of homologous gene sequences of organisms selected at random from the environmental sample. Using these data, biodiversity is quantified by counting clusters of sequences (operational taxonomic units, OTUs) with mutual genetic distance smaller than a prescribed threshold. Genetic distance is measured as the number of mutations relative to sequence length. With increasing threshold distance, the number of OTUs declines. Figure 1 summarizes typical relationships between genetic distance and OTU counts assembled from the literature (table 1).

**Table 1.** Datasets in figure 1. (CR1 and CR2 correspond to the combined counts for nematoda and ‘other Eucariota’ [10], for CR1 covering only the sites Littlehampton 2 and 3. FO-O and FO-E were derived from the raw data for Littlehampton 1 by different methods. A description of OCTUPUS can be found in the appendix of [11].)

label	organisms	habitat	approximate body size	algorithm to count lineages or OTUs	reference
CR1	Eucariota	marine littoral benthos	45–1000 µm	OCTUPUS	[10]
CR2	Eucariota	tropical rainforest	45–1000 µm	OCTUPUS	[10]
FO-O	Eucariota	marine littoral benthos	45–1000 µm	OCTUPUS	[11]
FO-E	Eucariota	marine littoral benthos	45–1000 µm	ESPRIT	[11]
JO	*Astraptes fulgerator*	tropical forests	5 cm	MOTU	[12]
PO	*Rivacindela*	arid Australia	1–2 cm	maximum-likelihood phylogeny	[13]

A remarkable common finding of these analyses is the steady decline of OTU counts with increasing threshold values, making it difficult to determine a ‘correct’ value of genetic distance that would allow the identification of OTUs with genetic species and of OTU counts with species richness. Put simply, the data appear not to support the existence of well-defined genetic species: a result at odds with much of the existing theoretical literature. Here, we report on numerical and analytic studies conducted to clarify under what conditions ecological species should form, and what, for empirical data such as in figure 1, the signature of a breakdown of the formation of ecological species would be.

## 2. Model

We study an individual-based stochastic model of competing organisms which reproduce with mutation. It has the following structure and dynamics.

### (a) Organisms

Each organism is entirely characterized by a quantity which determines its role in the community, for example, its traits in variant I of the model or its genome in variant II.

In the simple phenotypic variant I of the model we will consider here, the organisms will have a single trait, so that each individual in the model is entirely characterized by an ecotype *x*, where *x* is a real number. In the genotypic variant II, each individual is characterized by an *N*-dimensional vector of ones and zeros, with each entry of the vector being interpreted as distinguishing two viable alleles.

### (b) Population

At time *t,* there are *M*(*t*) organisms in the community. These will be specified either by their ecotypes *x*_1_, … , *x_M_*_(*t*)_ in variant I or by *N*-dimensional vectors ***x***_1_, … ,***x****_M_*_(*t*)_ specifying their genomes in variant II.

In order to define the processes of competition and reproduction, we need a criterion which specifies how close two individuals are to each other. This ‘distance’ between individuals *i* and *j* will be taken to be the distance between their ecotypes, *D_ij_* = |*x_i_* − *x_j_*| in variant I. In variant II, *D_ij_* will be the number of sites at which the genomes of the two individuals differ (the Hamming distance).

### (c) Birth

Each organism reproduces asexually at the same rate, and we choose units of time so that this rate equals unity. The characteristics of the offspring are chosen probabilistically according to a distribution centred on the parent, but falling off with distance *D_ij_*. In variant I, the ecotype of the offspring is chosen from a normal distribution with variance *μ* centred on the ecotype of the parent. In variant II, each of the *N* loci will independently mutate with probability *μ*.

### (d) Competition

The strength of competition between two individuals *i* and *j* is taken to be a function *g*(*D_ij_*), known as the competition kernel, which decreases with *D_ij_* to reflect increased competition between similar individuals. The total competition experienced by individual *i* is given by this competition strength summed over all other individuals divided by *K*, the carrying capacity. The total death rate is the sum of this total competition term, and some natural death rate that depends on the ecotype or genotype of the individual.

Various functional forms for the dependence of competition on distance *D_ij_* have been studied, for instance, a normal distribution with standard deviation *w* or a ‘top-hat’ which is a non-zero constant only when |*D_ij_*| < *w*. The mechanism that leads to the clustering that we describe in §3 is very robust and independent of the precise model specification, and thus we have limited our study to the two variants mentioned, with specific choices for the competition function and the nature of the offspring produced.

### (e) Niche fitness

In variant I of the model, extreme phenotypes (very large or small values) suffer a fitness penalty. We model this by adding a term 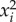 to the death rate of each organism. In variant II, all genotypes are (in isolation) equally fit, meaning that death rate is entirely determined by competition.

The model specification for both variants is summarized in table 2. Pseudo-code detailing the algorithm used is provided as the electronic supplementary material. We have chosen to investigate variant II in addition to the more standard variant I for three main reasons. First, for models of type I artefacts are known to occur which are attributable to the simple mathematical structure of this class of models [14–17]. Second, the restriction of ecotypes to a single niche axis might be a constraint, inhibiting the formation of species. Finally, because variant I is not an explicit genetic representation, it is not clear how the criterion for the formation of ecospecies derived below could be interpreted at the genetic level.

**Table 2.** Summary of the model definitions for variant I and II. (In both cases the only parameters are the carrying capacity *K*, mutation strength *μ* and competition kernel *g*.)

	variant I	variant II
genotype/phenotype	real number, e.g. *x* = 0.45	binary vector, e.g. ***x*** = (0,1,1,1,1,0,0,1)
similarity measure	*D_ij_* = |*x_i_*−*x_j_*|	*D_ij_* = number of elements ***x**_i_* and ***x**_j_* have in common
birth rate	*b*(*x_i_*) = 1 for all *i*	*b*(***x**_i_*) = 1 for all *i*
effect of mutation	add a Gaussian random number of variance *μ*	each bit independently flipped with probability *μ*
death rate	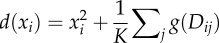	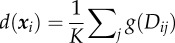

Having specified the model, we now go on to describe the formation of clusters of individuals which is seen as the system dynamics evolves in time. This is illustrated using numerical simulations, but analytic results are also available which give criteria for cluster formation [8,9].

## 3. Phylogenetic and metagenomic signatures of species formation

**Figure 2.**
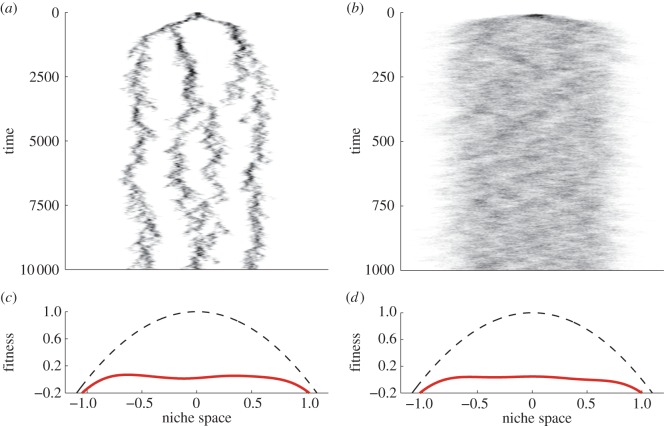
Sample model output in variant I. (*a*,*b*) illustrate the density of model individuals along the niche axis through time, starting with a single individual at *x* = 0. Model parameters are *K* = 10^3^, *g*(*x*) = (2*πw*^2^)^−1/2^ exp[–*x*^2^/(2*w*^2^)] with *w*^2^ = 0.1, and mutation rates *μ* given as 10^–5^ for (*a*) and 10^−3^ for (*b*). In (*c,d*), (red) solid lines are the corresponding invasion fitness landscapes in the final states of the simulations, the (black) dashed lines indicate invasion fitness for an empty community for comparison. (Online version in colour.)

We begin by presenting some typical outputs of variant I of the model, to illustrate the basic concepts and results. Recalling that the mean population growth rate for a birth–death process with birth rate *b* and death rate *d* is given by *b* – *d* [18], one can compute a fitness landscape for this model as *f*(*x*) = 1 − *d*(*x*), where *d*(*x*) is the death-rate profile defined in table 2. For a habitat containing no individuals at all, the fitness landscape is given by the inverted parabola *f*(*x*) = 1 − *x*^2^ indicated in figure 2*c,d* (dashed lines).

Figure 2*a,b* presents outputs of two model runs differing only in the value of the mutation rate *μ*. For small *μ*, the species descending from the single initial individual splits into several ecological species that remain separated in niche space. Very similar results have frequently been presented in the literature [19]. For larger *μ*, distinct species do not form, or form only during the initial transient phase. Later, phenotypic variability within species increases, and, at least visually, species associated with distinct niches can no longer be identified.

Note that the species present in figure 2*a* do not appear to occupy locations of maximal fitness. The theory of adaptive dynamics would suggest that these species are therefore unstable and will either move or undergo sympatric speciation. This does occur in long simulation runs, however, the system never reaches an equilibrium state. Instead, the long-time dynamics is characterized by species clusters which move and interact, without ever becoming optimally fit. The state of the system at the end of the simulation presented in the figure is typical of a snapshot of a long simulation run. By contrast, the higher rate of mutation in the simulation shown in figure 2*b* allows it to reach something of an equilibrium, in which organisms are spread almost uniformly throughout niche space. In both simulations, the emergent fitness landscape is very much flatter than that for the empty habitat.

This emergence of a nearly neutral fitness landscape needs to be taken into account to understand the mechanism underlying the transition between the scenarios of small and larger mutation rate. In previous analytical treatments of the model [8], we identified two regimes of species formation: a strong regime in which quasi-stable clusters emerge, and a weak regime in which there is some noise-driven clustering but no persistent species formation. The transition between these regimes can be understood by considering the neutral formulation of the model in which the competition kernel is flat and there is no niche-specific fitness. In this case, it was found that phenotypic variability, i.e. the standard deviation of *x_i_* within a lineage, is determined entirely by the magnitude of *μK*, the product of mutation rate and carrying capacity.

This result has important consequences for understanding the relationship between carrying capacity and mutation strength in the general case of an arbitrary competition kernel *g*. For fixed *K*, as *μ* increases, phenotypic variability will eventually become as large as the niche width *w*, and so the clear association of lineages with their respective niches disappears. This is the source of the difference between the plots in figure 2. Crucially, the same relationship holds if mutation strength is fixed and carrying capacity is varied [8]. This understanding allows us to interpret our simulation results also for situations expected to arise with large population sizes, which are difficult to simulate. Because, under fitness neutrality, any increase in the value of carrying capacity *K* is effectively equivalent to an increase in *μ*, the model suggests that for organisms occurring in large abundances the scenario without ecological species can arise even when, at individual level, mutations are small compared with niche width.

**Figure 3.**
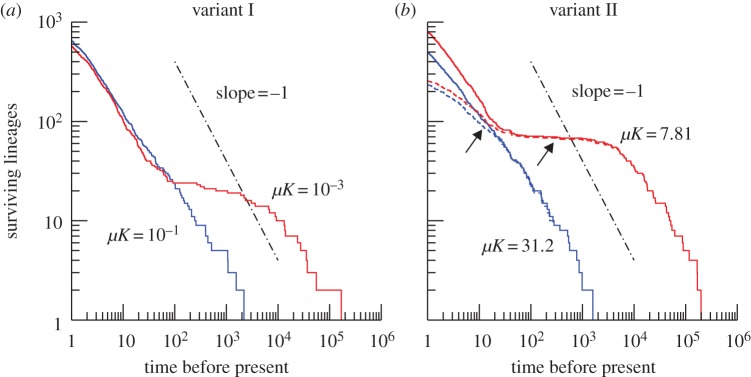
Phylogenetic and metagenomic signatures of ecospecies formation. Simulation outputs with *μK* = 10^−1^ and *μK* = 10^−3^ in (*a*) and *μK* = 31.2 and *μK* = 7.81 in (*b*), as labelled. Competition kernels were normal with width *w*^2^ = 0.01 in (*a*). In (*b*), it was of top-hat type with width *w* = 10, and the binary vectors ***x*** distinguishing genomes had length *N* = 32. Dashed lines correspond to subsampling of 750 individuals. Arrows mark approximate locations of inflection points of the upper (red) dashed line, corresponding to *μK* = 7.81. Dash-dotted lines represents the scaling relation (lineages)∼1/(time) for comparison. (Online version in colour.)

As well as the changes in phenotypic variability associated with *μK*, there are profound alterations to the structure of the organisms' ancestral tree. These changes are best presented in a lineages-through-time (LTT) plot, giving the number of branches of the ancestral tree at each point in time before the present. In figure 3*a*, we show LTT for variant I of the model with *μ* = 10^–4^ and *μ* = 10^–6^, with fixed carrying capacity *K* = 10^3^. Care was taken to run the simulation for sufficiently long to remove any effects of the initial condition, which avoids complicating interpretation of results by the phenomena occurring during the initial transient phase of dynamics [20].

Considering the case of small mutation rate (*μK* = 10^–3^) and going backwards in time, the number of lineages initially declines rapidly until for each ecospecies only a single lineage remains. Over intermediate timescales, this number remains essentially constant at a value corresponding the species richness supported by the habitat (≈22). However, over longer times spontaneous speciations and extinctions occasionally occur, so that the number of surviving lineages declines further. Characteristic of the formation of species is the plateau in the LTT plot, which separates the timescales of the intraspecific and the interspecific phylogeny. As the value of *μK* increases, the distinction between these two timescales is increasingly blurred. When the intermediate plateau disappears (*μK* = 10^−1^), a distinction between intraspecific and interspecific phylogeny becomes impossible.

The conclusions drawn from variant I of the model can be summarized as follows: first, the emergence (or not) of ecospecies is controlled by the value of *μK*, meaning that very large carrying capacities can be expected to suppress the formation of species; second, this effect is visible in LTT plots, which exhibit a characteristic plateau in the event of species formation. We must now ask whether these findings are general or merely an artefact of the simple one-dimensional niche space formulation, and whether we can draw a useful comparison with data gathered from the field.

Variant II of the model helps answer these questions. The reformulation of the model in high-dimensional, discrete, genetic space is a major change. However, our theoretical analysis once again shows that the variability (this time understood in a genetic sense) of the population is controlled by *μK* [9]. Moreover, the LTT plots produced by simulations of the genetic variant of the model show the same characteristic plateau for low *μK*, separating intraspecific from interspecific phylogenies, which vanishes as either *μ* or *K* is increased (figure 3*b*). The LTT plots in the genetic variant are particularly useful as they give the first opportunity to compare (qualitatively at least) with field data. Our trees are constructed as lineages of individuals, however, counts of lineages of species are identical for times longer than within-species coalescence times [21]. The taxonomic units in figure 1 are clusters of gene sequences of individuals that are summarized into units for the sole reason of low genetic distance. Under the standard assumption that genetic distance is proportional to the time since the most recent common ancestor, numbers of clusters smaller than a given radius are equivalent to numbers of lineages at a given time in the past. The question is thus whether the LTT plots produced by the model resemble OTU counts from biodiversity surveys.

When comparing these graphs with empirical data, one needs to take into account that in the empirical analysis only subsets of the individuals forming a community are sampled. The number of observed surviving lineages obviously cannot exceed the number of individuals sampled, whereas the true number of lineages surviving over a short time period is much larger. The simulations from which the curves in figure 3 are derived typically contained around 1500 individual organisms, and we have implemented random subsampling of 50%. Crucially, the modified LTT plots for the scenario with ecospecies (low *μK*) contain two inflection points (changes in the direction of curvature indicated by arrows in figure 3*b*). One at the centre of the plateau corresponding to community species richness, the second corresponding to the onset of the effect of subsampling on short timescales. Between very short and very long times, the slope of the double-logarithmic LTT plot therefore first increases, then decreases and then increases again.

In view of these model results, it is noteworthy that two of the empirical LTT plots in figure 1 exhibit the pattern we find for the regime with ecospecies. For both the cryptic species complex of *Astraptes fulgerator* butterflies and the genus of Rivacindela beetles, the slope of the double-logarithmic LTT plot first increases, then decreases, and then increases again. Interpreting the second inflection point as an imperfect plateau corresponding to coexisting species, species richness in both complexes can be estimated from the corresponding numbers of OTUs approximately as 10–20. We note that with 1–5 cm body size, both kinds of organisms are comparatively large.

By contrast, none of the four double-logarithmic LTT plots obtained from meiofauna exhibits pronounced inflection points. The curves have slopes around −1 and are bending slightly downwards, similar to what we found in the simulations for the scenario without ecospecies (figure 3*b*, lower (blue) dashed line, corresponding to *μK* = 31.2). Possible explanations could be that the inflection points corresponding to ecospecies in these cases lie either at very small (≲1%) or very large (∼10%) genetic distances, and are therefore not covered by the LTT plots, or are simply methodological artefacts. Another explanation could be that for the meiofauna sampled in these studies ecospecies cannot form, because, as for our model simulations, *μK* in these cases is too large.

## 4. Discussion

In numerical and analytic investigations of the model, we found transitions from states with ecospecies for *μK* very small to states without ecospecies when *μK* is large. This phenomenon can be understood intuitively as the combined effect of three mechanisms: (i) for neutral evolution, the intraspecific variability of the phenotype owing to mutations accumulated over generations increases with (effective) population size [22], (ii) nearly neutral fitness landscapes naturally emerge from evolutionary processes, even without strong intraspecific variability (figure 2*a*; [23]), and (iii) any increase in intraspecific variability of the phenotype will further smooth out the fitness landscape. The three mechanisms thus reinforce each other. If the resulting phenotypic variability is larger than niche width, then ecospecies cannot be distinguished.

Within this framework, formation of ecospecies requires that population sizes are sufficiently small that, despite approximate neutrality, intraspecific variability of the phenotype remains small compared with niche width. The mathematical derivation of adaptive dynamics implicitly assumes this last condition to be satisfied, as it makes use of the limit of nearly faithful reproduction, which means that the emergence of mutants is rare on population-dynamical timescales [19]. Crucially, this condition may not be satisfied for small organisms that form large populations.

Because the biomasses supported by dominant populations do not change much with body size [24,25], smaller organisms tend to have numerically larger carrying capacities *K* and are therefore less likely to form ecospecies than larger organisms. An approximate rule of thumb for the body-size threshold above which ecospecies will easily form can be obtained by evaluating the product *μK* for a range of different taxa. The mutation rate *μ* appearing in variant II of our model is directly comparable to naturally occurring rates of synonymous mutation, estimated to be around 10^−10^ to 10^−9^ per locus per generation for many different taxa [26]. For the carrying capacity *K*, we use global population estimates. While it is clear that geographically separated individuals do not experience direct competition with each other, it has been observed that the dispersal of small organisms happens on a very much shorter timescale than their evolutionary dynamics [27,28], meaning that competition between lineages is largely independent of space. Alternatively, one may think of geographical location as simply another inherited trait, and dispersal as analogous to very high rates of mutation, meaning that populations may be relatively homogeneous in geographical space, but clustered in niche space.

**Figure 4.**
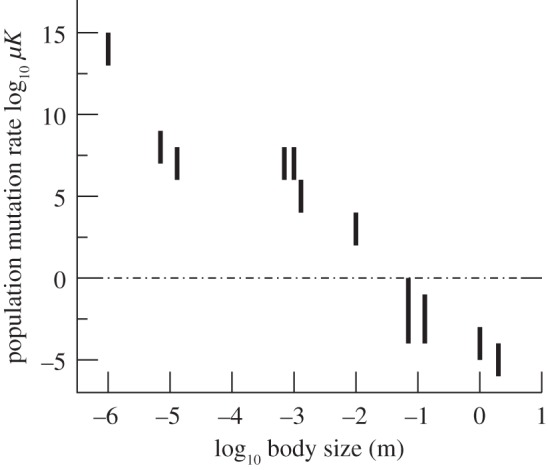
Estimation of the body-size threshold for ecospecies formation. Vertical bars correspond to ranges of population mutation rates per generation according to literature data [26] for a range of taxa. These are, from left to right: bacteria, marine diatoms, dinoflagellates, planktic foraminifers, bryophytes, benthic foraminifers, insects (beetles, *Drosophila*), lizards, rodents, turtles and crocodiles, and carnivores.

In figure 4, we show the result of combining data on published population sizes (as upper bounds on the number of competing individuals) and speciation rates assembled for various taxa in [26] with our own order-of-magnitude estimates for adult body sizes. Despite substantial methodological uncertainty, the data in figure 4 do give a clear idea of the orders of magnitude involved. As a function of body size, the transition between the strong and weak species formation regimes appears to occur at around the scale of centimetres to millimetres (taking into account that effective population sizes can be a few orders of magnitude smaller than actual ones [29]).

Interestingly, the empirical LTT data we assembled in figure 1 exhibit patterns similar to those we would expect in the presence of ecospecies only for organisms that have body sizes measured in centimetres, but not for meiofauna with much smaller body sizes. This suggests the millimetre scale as the approximate region in which the ecospecies concept begins to break down. One needs to be aware, however, that the few empirical studies underlying the data shown in figure 1 are too inhomogeneous in the protocols used for sample collection, sequencing and data processing to support any strong conclusions. Moreover, systematic and statistical errors can lead to inflated estimates of OTU counts from pyrosequencing [30]. It is possible that more systematic studies of the dependence of the structure of phylogenetic or metagenomic data on body size will reveal that the correspondences with our simulated data (figure 3) are accidental. Being conservative, all we can say at this stage is that the data currently available to us are consistent with the hypothesis that indeed ecospecies form only for organisms with body sizes exceeding the millimetre scale.

Our model is by necessity a highly simplified representation of reality. Among various possible shortcomings, the most relevant to the work presented here is that only asexual reproduction is considered. Drawing on past results [6,22], we expect that modifying the model to include sexual reproduction may alter the details of the speciation mechanism, but should not overturn the basic dependence on *μK*.

The question as to whether small multicellular organisms do form ecospecies or not unquestionably deserves further study. Small organisms make large contributions to both biodiversity and ecosystem functioning. Despite alternative metrics having been proposed [31], species richness is still a widely used metric in biodiversity censuses. In view of the known weaknesses of other criteria for partitioning phyla into species [32], a breakdown of the ecological species concept might mean that species richness is indeed not a well-defined quantity, calling for the use of species-independent metrics. New challenges would arise also for efforts to model the interactions between organisms and populations in ecological communities. The vast majority of community models built on the assumption that species as discrete ecological units exist. New conceptual frameworks would be required if this paradigm cannot be upheld.
